# Strategies for achieving efficiency in the general practitioner’s everyday life

**DOI:** 10.1080/02813432.2022.2097609

**Published:** 2022-07-19

**Authors:** Kok-Wing Giang, Josefine L. Lilja

**Affiliations:** a Research, Development, Education and Innovation, Primary Health Care, Region Västra Götaland, Götaland, Sweden; b Department of Psychology, University of Gothenburg, Gothenburg, Sweden; c General Practice/Family Medicine, School of Public Health and Community Medicine, Institute of Medicine, Sahlgrenska Academy, University of Gothenburg, Gothenburg, Sweden

**Keywords:** Clinical practice, clinical competence, communication, family medicine, patient-centered care, general practitioners, general medicine

## Abstract

**Objective:**

To describe concrete, effective strategies used by experienced GPs to achieve time efficiency, increase patient satisfaction and maintain high medical quality during patient meetings.

**Design:**

Qualitative observation yielded field notes for qualitative content analysis according to Graneheim and Lundman. Follow-up telephone interviews were conducted to get feedback from patients.

**Setting:**

A normal working day with patient meetings in a primary health care center in Sweden.

**Subjects:**

Five GPs known for being experienced and well-functioning clinicians were strategically chosen to participate in an observational study during patient meetings. Afterwards a random selection of 25 patients (five from each GP) were asked to rate their experience of their meeting.

**Results:**

Observation and analysis of GPs’ work before, during, and after patient meetings revealed several concrete strategies, which we classified into two main categories: *Behavioral* and *Communicative,* comprising nine and seven *s*ubcategories, respectively.

**Conclusion:**

Most important behavioral skills for time efficiency were a GP’s ability to handle interruptions, and effective administration. Medical quality during patient meetings was most supported by GP continuity and relationship, an exploratory patient-centered approach, a focus on one task at a time, and the ability to acknowledge and learn from medical uncertainty. Patients were most satisfied with GPs who had good communicative skills, good GP continuity and relationship.

**Key points**
- The changing field of general medicine requires general practitioners (GPs), to work efficiently, but studies on effective work strategies for GPs are scarce.
- GPs used several concrete strategies falling into two broad categories (behavioral and communicative) that may also be important for other practitioners wishing to improve their methods in clinical patient work.
- The most important strategies for time efficiency were mainly behavioral; for medical quality during patient meetings, a mix of behavioral and communicative; and for patient satisfaction, communicative.

## Introduction

Primary care, defined as medical care provided outside the hospitals is characterized by its holistic view; main responsibility, proximity, and accessibility to the patient; continuity and quality of care; and safety and collaboration [1]. Primary care in Sweden is provided by local primary health care centers (PHCs) by general practitioners, nurses, psychologist and social health workers. General practitioners (GPs or family doctors) give basic medical treatment that does not require the technical resources or special skills at a hospital [2,3].

GPs’ tasks include diagnostics, treatment, counseling, rehabilitation, and disease prevention. To offer patients the best possible primary care, GPs require both a broad knowledge of general medicine and specific competencies [4]. Verbal behavior and patterns of consultation skills of a GP were stressed and studied already in 1976, comparing different communication styles among GPs in Britain [5].

As medical tasks are increasingly transferred from hospitals to PHCs, GPs’ administrative requirements, along with added increasing guidelines, care programs and standardized care process and other duties also increase, usually faster than existing resources and at the expense of clinical patient work. It is becoming more common for GPs to work under high stress caused by assigned tasks they deem unnecessary or unreasonable [6,7] and disruption of their focus, which can result in errors and missed or forgotten information [8].

Although it is not entirely known how many GPs are actively working in Sweden. For PHCs to provide adequate care, the Swedish Medical Association recommends a patient-GP ratio of 1,500:1; since the year 2000, the Swedish Parliament has also supported this goal [9]. In 2012, the 4,784 full-time GP positions in primary care provided a patient-GP ratio of 1,942:1. To reach the target ratio, an additional 1,400 full-time positions are needed [9–11]. Meanwhile, this staffing shortage leads to increased burnout in existing GPs [1,6].

Expectations on GPs have also grown, as patient satisfaction has become increasingly important, especially since 2008, when patients were allowed to choose their own PHC [12]. Since the rise of the internet and widely available healthcare and medical information, patients are better informed and more equipped to challenge their GPs’ assessments, explanatory models, and treatment choices. Patients are also expected to be involved in decisions about their care [13], while GPs are expected to focus on the patient as a person, to build trust, communicate successfully, and reach agreement [14]. Close and continuous relationships between patient and GP facilitate communication and increase job satisfaction as a GP [15]. In a focus study, describing the challenge of patients with problems and symptoms related to medically unexplained symptoms (MUS), further emphasized the GPs communication skills [16], which was also valued highly by patients, more than availability [17].

To improve communication with patients, GPs are taught the PRACTICAL consultation technique [18]. The patient meeting is divided into three parts: (1) the patient’s part, focused on *Prior, Relationship*, and *Anxieties*; (2) the doctor’s part, addressing *Common understanding, Translating, Interaction*; and (3) the common part, focused on *Converting insight, Agreement check,* and *Let's try it*. By sticking to this communication structure, according to the author, the patient meeting can be shortened to about 15 min [18,19]. This model has given rise to a simpler, yet still useful, guide to initiating the consultation called Five Cards. The patient part is described in more detail as a game in which patients have three cards: *Thoughts*, *Concerns*, and *Wishes*, and GPs have two: *Receipt* (emotional validation) and *Summary* (summarizing what the patients have said). During the patient part, GPs play their doctor cards as necessary to encourage the patient to play all three of theirs before moving on to the doctor’s part with more specific questions about the current symptoms followed by a physical examination. The common part completes the consultation with a discussion in which the GP collaborates with the patient to agree upon a treatment plan [20].

Another conversational technique that GPs use is motivational interviewing (MI), using reflective listening to show empathy and guide patients toward insight into current behaviors that contradict their own goals or values. At the same time, they try to avoid arguments and confrontation, to adapt to the patient’s resistance rather than oppose it directly, and to support optimism and self-efficacy [21].

An ethnographic study [22] shed light on how GPs’ personal styles combining spontaneous actions and intentional strategies affect their choices about how to handle certain parts of the clinical work. Both parts are important to a competent GP. A qualitative group interview study [23] revealed a number of effective working behaviors in areas such as preparation for the patient meeting, focus on the current problem, time management, confirmational physical examination, collaboration with other professionals, and record keeping as an aid. Patterson *et al.* [24] also emphasized certain characteristics of a GP that are considered particularly important such as empathy, communication skills, and clinical knowledge.

Effective time management strategies such as setting short- and long-term goals, prioritizing responsibilities, planning and organizing activities, and minimizing “time thieves” could increase GPs’ productivity and limit burnout [25].

Considering GP staffing in a PHC as a static factor, even if only temporarily, allows the possibility that the GP’s way of working is a factor that can change. The PHC’s work constantly changes as new challenges emerge, but (to our knowledge) few studies describe how experienced GPs work or what specific strategies contribute to their time efficiency, patient satisfaction, and quality of care.

### Purpose of the study

The purpose of the study was to investigate, specify, and concretize examples of behavioral strategies used by well-functioning experienced GPs during a clinical working day and to analyze them in terms of time efficiency, quality of care (mainly medical quality related to patient meetings and clinical work), and patient feedback.

### Scientific questions

What strategies do GPs use that contribute to time efficiency, patient satisfaction and good quality of care? (Quality of care in this context is defined as: a presenting medical problem getting an adequate clinical response from the clinician, in this case the GP in question.)

Do GPs use any specific consultation or conversation techniques? Were the patients satisfied with their meeting with their GP, and what made them feel that way?

## Method

### Study design

We conducted a participatory observational study of working strategies in a strategic selection five experienced GPs’ during a normal day at work with patients. The resulting field notes were later analyzed using the qualitative content analysis of Graneheim and Lundman [26] and followed by telephone interviews collecting patient feedback.

### Participants

Experienced GPs were needed to investigate good behavioral strategies. We asked colleagues in the nearest collegiate medical network, GPs/managers of PHCs, and resident physicians specializing in general medicine to recommend skilled GPs who might consider participating. They suggested a total of 13 experienced (at least 10 years) GPs, who were all known for being well-functioning, both in the sense of being a helpful leader, working well with other staff groups and fulfilling their role well as a diagnostician and medical advisor for the patients. Due to the observer changing workplace from one private PHC to another private PHC, it was pointed out by management not appropriate for some GPs to participate in a study conducted by a doctor affiliated with another private PHC. GPs from public and other private PHCs were thus excluded from the study. Another GP declined due to time constraint, leaving five participants who agreed to take part in the study. The five participants were clinically active GPs, two women and three men, between 48 and 67 years old, with between 10 to 35 years of experience working as GPs in primary care. During the study they all worked in well-established, well-functioning private PHCs in and around Gothenburg.

The patients included in this study were adult patients between 30 and 70 years old, who understood Swedish enough to understand the nature of the study.

After the observation, the observer randomly chose five patients from each GP to take part in a follow-up interview. 13 of the 25 chosen patients answered, five women and eight men. They were asked to rate their experience of their GP with a short questionnaire over the telephone. The other 12 patients were excluded from the study, after another five attempts to reach them were made, during a two-week period.

### Ethical considerations

A fact sheet and consent form were sent to the GPs *via* email prior to the observation. Before the meetings, each patient was informed orally and *via* an information sheet and a consent form. All GPs and participating patients signed their written consent, including names and telephone numbers for the patients.

Consideration was made about the presence of the observer, theoretically could affect the doctor with added peer pressure and feeling of surveillance, and the patient with the false feeling of extra security by having another doctor listening in the room.

This study was approved by the Regional Ethics Review Board in Gothenburg (registration no.: 201902379).

### Data collection and analysis

The observations were conducted on clinical workdays selected in consultation with each GP. The observer followed the GPs throughout their working day and used a prepared observation schedule to keep detailed field notes of how they performed their clinical work. Before each meeting, the observer noted GPs’ preparations, informed the patients about the study, and collected patient consent forms, sometimes with the help of the receptionist. The observer continued to keep field notes throughout the meeting. After the meeting, the observer noted how the GP conducted follow-up work and began to prepare for the next patient. The time spent on each part of the patient meeting was also recorded. The notes were then transcribed for analysis. To maintain transparency, before the analysis, each GP was given their part of the transcript to confirm, comment and possibly add to or remove, the recorded information. All transcripts were confirmed without any changes.

Graneheim’s qualitative content analysis [26] was applied to the transcribed field notes to analyze behavioral strategies recorded during the observations: 1) All transcribed text from the five observations was placed in the same Excel file. The text was read several times to get an overall feeling of the whole context. Data from each GP was coded by color and all text from each GP were placed. 2) The text was arranged into smaller meaningful paragraphs. 3) These meaningful paragraphs were condensed into a description close to the text. 4) The underlying information was interpreted. 5) The condensed paragraphs were coded, and subcategories and categories were selected. 6) The entire analysis table was sorted alphabetically by subcategory.

Each GP’s average time spent per patient including the time spent in all stages: before, during and after the patient meeting, was calculated as (total number of minutes allocated to patients throughout the day of observation – minutes of interruption)/(total number of patients met during the observed day). The five GPs were assigned letters to distinguish them.

Following analysis of the observations, 25 randomly chosen patients (five from each GP), were asked to rate their experience with their GP in a follow-up telephone conversation. A short questionnaire asked them for their positive and/or negative feedback and their reasons.

The observer designed the study, collected the data, and conducted the analysis in consultation with the supervisor, and the observer and supervisor continuously co-validated the results for additional credibility.

## Results

The analysis of the data collected resulted in two main categories of strategies: *Behavioral* and *Communicative*, comprising nine and seven subcategories, respectively (Table 1). These subcategories are presented relative to the stages of patient meeting they were observed: before, during, or after the meetings.

**Table 1.** Division of subcategories.

Behavioral strategies	Communicative skills
Preparation	Conflict management
GP continuity and Relationship	Investigation and Exploration
Interruption management	Authority
Focused physical examination	Information
Follow-up	Validation
Technology	Strengthening
Administration	Collegial information exchange
Knowledge of the health care system and the flow process in health care	–
Self-improvement	–

### Before the patient meeting

There were some influencing factors in this “before stage” leading to the actual patient meeting, which could help determining efficiency during the meeting. Different preferences in preparation, if the patient was known by the GP, and if the relation between them were good, could all influence the efficiency of the patient meeting.

#### Preparation

GPs differed in their preparation before a patient meeting. Some preferred to spend more time preparing by carefully reading the patient’s medical record and taking notes before entering the patient meeting, while others preferred to meet the patient first before looking at the medical record.

#### GP continuity and relationship

In some patient meetings where the GP seemed to know the patients in advance, fewer initial open-ended questions were observed during the meeting; often the GP went straight to the point and asked short closed-ended control questions to find out the problem. It seemed that previous knowledge of the patient in question also led to shorter patient meetings. In sum, a previously well-established relationship, based on reliability and care, seemed to facilitate communication between the GP and the patient.

### During the patient meeting

This stage refers to the time between the GPs first engagement with the patient and until the meeting ends either by the patient or the GP leaving the room. Communicative skills and physical examination skills dominate at this stage. GPs adapted their communicative styles to the situation by using validating, strengthening, informative, authoritarian, or exploratory strategies expressed through body language (posture, sitting position, and hand gestures), tone of voice (especially emphasis on important words), and managing language barriers by using shorter sentences and simpler words, speaking more slowly, or switching from Swedish to English.

#### Conflict management

When a patient made a request that the GP found unreasonable, GPs tended to use validating and strengthening arguments along with information to resolve the conflict.

#### Interruption management

GPs usually concentrated on doing and finishing one task at a time, but they almost always continued to talk to the patient during the physical examination, quickly shifting focus between the examination itself and interacting with the patient, asking questions, commenting on findings, and responding to the patient’s new questions and concerns as they arose. A mix between the patient’s part and the doctor’s part.

In another example of GP’s quick change of focus, some read the patient’s record during the medical interview, shifting between listening to the patient and finding information on the computer to guide further questions.

GPs were often interrupted by phone calls, internal messages, or knock on the door. Most dealt as quickly as possible with the interruption before continuing with their task before, during, or after their meeting with a patient.

#### Investigation and exploration

All GPs interpreted what patients told them and asked follow-up questions to get as clear a picture as possible of patient’s condition to facilitate a diagnosis.

#### Authority

Sometimes, when the meeting was coming to an end and the patient had more questions, raised new problems, or offered new information, GPs closed the meeting by shifting to a more authoritative style, giving a short answer or general advice.

#### Information

GPs informed patients of the results of their examinations and recommended or prescribed medication or other treatments. Sometimes, to ascertain that the patient understood the information, the GP could summarize and/or repeat the information given.

#### Validation

All GPs used validation in one form or another as a strategy during the patient meetings. They usually identified patients’ worries, concerns, thoughts, and wishes and responded to them with comments, sounds such as “hmm,” and/or changed body language, also “yes, yes”, “I understand” and longer sentences e.g. ”Then the question is: how is the best way for us to proceed with our medical investigation.” Some GPs also summarized and/or repeated what the patient had said as confirmation that they had heard the patient’s concerns.

#### Strengthening

Some GPs responses seemed formulated to confirm what patients said and to mentally strengthen them. For example, one GP who agreed to a patient’s request for blood pressure measurement said, “Actually, this is a perfect blood pressure,” stressing the word “perfect.” Other GPs empathized with the patients (e.g. “If I were in your situation, I would…”).

#### Focused physical examination

All GPs conducted a physical examination focused on specific areas of the body depending on anamnestic information from the patient and/or additional discoveries during the examination.

### After the patient meeting

This stage refers to the time between the end of a patient meeting and the “before stage” of the next patient meeting. The time spent were largely emphasized by skills with the computer, administrative skills of journaling, knowledge of the health care system and finding the right people to ask for further advice on follow-up strategies, and efforts on self-improvement. Also, small breaks could occur when needed.

#### Follow-up strategy

Patients were usually responsible for any follow-up. They were asked to keep track of their symptoms and return for a new meeting with the GP if necessary. If the GP thought there was a medical reason for follow-up within a certain period of time, they arranged the appointment themselves and the patient received a reminder note of the new appointment before leaving the PHC.

#### Technology skills

All GPs had broad and deep knowledge of the computerized medical record system and the associated administrative tasks. GPs who dictated patient records often kept their summaries limited to the background, current situation, current examination status of the patient, and their own assessment. Some GPs used a computer in the examination room for quick access to information on the patient during the meeting.

#### Administrative strategy

Each GP tried to use breaks between patient appointments to attend to administrative tasks such as documenting patient records. They usually chose to do those tasks immediately, but would postpone them if they were interrupted or they expected them to take more time than they had at the moment.

#### Knowledge of the health care system and the flow process in health care

The GPs showed extensive knowledge of how the health-care system worked in their specific PHC. They knew who did what and who was responsible at the PHC level and at levels beyond their own clinic.

#### Collegial exchange of information

The GPs were seen to consult with each other on various issues during the day. Some PHCs facilitated this consultive environment by placing GPs in an open office landscape with easy access to each other and separate examination rooms for patient meetings. GPs in other PHCs with separate offices had an open-door policy to welcome colleagues’ questions.

#### Self-improvement

GPs demonstrated a deliberate effort toward self-improvement, such as reflection and self-analysis after patient meetings. GPs often discussed with their colleagues what they should do next, and how. None of the GPs were afraid to ask colleagues for help, and some openly acknowledged their shortcomings and asked for better ideas of how to deal with a particular problem.

When uncertainty arose or when mistakes were discovered during the patient meeting, GPs immediately acknowledged them to the patient and often apologized before continuing as usual.

### Time efficiency

Each GP was usually able to handle a patient meeting within 30 min, which is considered reasonable, but the time varied depending on the patient’s reason for the visit [19,20]. See (Table 2).

**Table 2.** GPs’ average time spent per patient.

GP	Time
GP A	12 min
GP B	27 min
GP C	23 min
GP D	30 min
GP E	20 min

### Patient satisfaction correlated with the strategies

The five GPs were assigned letters to distinguish them. GP A used a combination of authority, information, and validation. Of the four GP A patients who participated in the phone interview, three gave a positive rating, based mainly their history with the GP. They said it was easy to get an appointment with GP A, they were happy not to have to repeat themselves because GP A already knew their medical history, and they trusted GP A completely. The fourth patient gave a negative rating, citing a feeling of stress in the meeting and a lack of opportunity to ask questions. This particular situation corresponded to the observation of the patient being stiffer in their expression and seemingly a feeling of subtle unease.

GP B was usually well prepared, readily admitted any uncertainties, responded to patients informatively, and focused on strengthening and validating the patients. Of the two patients agreeing to the interview, one gave B positive rating based on feeling safe having known the GP a long time. The other gave B a negative rating, having expected the GP to have done more to investigate specific stomach problems and make a referral to a specialist, rather than saying to wait and return if the problem worsened.

GP C used an informative authoritative approach, sometimes using personal examples and often using body language such as hand gestures and validating the patient with short words of acknowledgement and nods. The only patient of this GP to participate in the telephone interview said that he trusts his GP, who naturally knows best, to decide everything about his treatments and that he would naturally do “what the doctor says.”

GP D’s style cycled through authoritarian, informative and validating strategies. D adjusted the language based on the patient’s needs and was especially good at picking up on and responding to patients’ worries. Four of D’s patients agreed to participate in the interview. Two were happy, one saying that their personality seemed to fit with the GP’s, and the other saying they felt that they had some say in their treatment. The other two, having different expectations, were less happy. One had wanted to be examined by a “specialist” and one said they would have received different treatment in their home country.

GP E maintained calm despite many interruptions and openly admitted any uncertainty, using validation and strengthening more often than information. The two patients who responded said that they felt the GP listened to them and was understanding of their situation and that the whole team did their best and gave quality service.

## Discussion

The purpose of the study was to investigate, specify and concretize the strategies used by well-functioning and experienced GPs during a clinical working day and to analyze them in terms of time efficiency and medical quality in conjunction with patient feedback. The data collected before, during, and after the patient meetings were grouped into two main categories of strategies: *Behavioral* and *Communicative*, comprising nine and seven subcategories, respectively.

Previous studies have described important characteristics and work behaviors of competent GPs [22–24]. Some of the behavioral strategies and communicative skills found in this study have been studied before, but as far as we know the specific behaviors that contribute to time efficiency, patient satisfaction, and quality of care have rarely been described. This study aimed to shed light on these behaviors in conjunction with patients’ feedback.

### Efficiency

The three behavioral factors that contributed most to time efficiency were GP continuity, interruption management, and administrative strategies. Continuity of care, knowing the patient before the meeting, seemed to have an impact on preparation time as GPs required less time to read the medical records of known patients and usually needed to ask fewer questions to discover the patient’s problem. GP continuity was also the basis of a good doctor-patient relationship that created trust, which reduced the time needed to motivate and persuade the patient about treatment options. Feedback from patients participating in the interviews further confirm that GP continuity was important for creating trust and satisfaction, facilitating in increased medical compliance.

All GPs used various strategies to manage interruptions and keep up with their administrative work. They most often preferred to complete one thing at a time, prioritizing the current task and avoiding or mitigating interruptions whenever possible. When necessary, however they could shift their focus between two different tasks, such as talking to the patient and performing a physical examination. At the same time, GPs had a positive attitude toward necessary interruptions by colleagues, nurses, and other staff and had an open door attitude or open office landscape. When interrupted or disturbed, the GPs under observation dealt with the additional stress by prioritizing and concentrating on one thing at a time to reduce the risk of error. It is possible that other GPs prioritize their own work over other staff’s problems and thus do not have an equally positive attitude to the interruptions. These GPs also showed good knowledge of both the local health care system and the administrative chores generated by a patient meeting, which could help to protect them against this type of stress. The observed GPs talked to patients while performing physical examinations and some shifted focus between the patient and the computer to obtain information more quickly and move the meeting forward. All GPs seemed to have a well worked out administrative strategy and used breaks between patients for other administrative work, reducing the administrative burden at the end of the day. Altogether these strategies seemed to create time-efficient and effective peer-to-peer consultations, also protect against increased stress and problems in the work environment. Based on these results, we recommend that PHCs discuss and encourage a common strategy and plan for collegial consultation.

### Medical quality

The most important behavioral strategies for medical quality were exploration, interruption management, and self-improvement. Exploring the patient’s agenda and symptomatology with follow-up questions to find out why the patient sought medical advice and to get as clear a picture of the disease as possible is a prerequisite for medical quality in the patient meeting. The clearer the picture GPs could get of the problem, the greater the chance they could understand the patients and make the right diagnosis, give the right advice, and prescribe the right treatment. Medical quality also includes the patients feeling that the GP listened to, and understood their situation, and trying their best to help, for trust to be formed. By doing “as the doctor says”, also increase the likelihood for better quality of care in a broader sense for the patient. Thus good doctor-patient relationship influence medical outcome [27].

GPs tried to perform one task at a time and to minimize interruptions, which previous studies have shown to increase the risk of errors [8]. Still, they tried to be helpful by being open to interruptions. Most of the GPs seemed unpretentiousness and were willing to admit uncertainty. Having the courage to ask for advice when needed may contribute to medical quality in the GP’s clinical patient work. As general medicine is an area of constant change and increasing challenges, competent GPs must continuously think about their working methods and come up with different ways to improve themselves and their workplace to maintain the necessary conditions for good medical quality. One way may be to raise these issues in collegial discussion groups and work together toward self-improvement by discussing patient cases and sharing the lessons learned.

### Patient satisfaction

Most patients pointed out the convenience of seeing the same GP and not having to repeat their medical history at every meeting, saving them from unnecessary frustration. They gave positive feedback about feeling heard, feeling their opinions mattered, and feeling they could trust and g*et al*ong with their GP, whose personality seemed to mesh with theirs. Patients gave negative feedback when they felt they had been rushed, discouraged from asking questions, and ultimately did not have their expectations met by their GP. It is also apparent that patients’ expectations varied widely. Different patients expected different behaviors from their GP, from always asking the patient’s opinion to taking a more authoritative approach and making decisions for the patient.

#### Communicative skills enhance patient satisfaction

GPs employed a broad spectrum of communicative skills ranging from authoritarian to informative and validating to strengthening. Communication styles depended on the GPs’ personal style, the kind of response they wanted to elicit from the patient, and the patient’s perception and expectations. Some patients seemed to think “the doctor knows best” and to prefer that the GP, as the authority, simply decide what to do. In these cases, GPs often gave patients specific information and encouraged them to take some responsibility and at least contribute to decisions about further treatment options. For other patients, GPs confirmed and validated the stories of those who seemed to want reassurance about their choice to see the GP and to know that they were listened to, e.g. “So you have had a sore throat for over a year. We need to take a look at that”. Several communicative strategies reduced patients’ anxiety, the most distinctive of which was validation, followed by strengthening, e.g. “You sound energetic today! Very good! Let’s do this!”. In one way or another, all GPs used validation by giving “receipts” and “summaries” of the Five Cards consultation technique [20] to confirm to patients that they had been heard and their symptoms taken seriously, e.g. “I understand, I understand! You have pulmonary embolism, meaning blood clots in your lungs. Poor you. That is tricky!”. This gave patients some peace of mind and the expectation that they would get the help they needed. Consistent with studies on motivational interviewing [21], the reinforcing response from the GP made patients feel mentally supported and confident that they were in control of their medical situation. The GP also reassured patients that everything would be fine. From an observational stand point, these patients seemed satisfied with the patient meeting. These ways of communicating fit well with a patient-centered approach [14], which has been described as an important element of GP competency [22–24].

GPs who demonstrated flexibility in their style also generated good patient meetings. This is well in line with studies on patient-centered approaches that emphasize the importance of exploring patients’ entire agendas and involving them in their own care, which ultimately increases patient satisfaction. We found in this study that GPs use several behavioral strategies and communicative skills to achieve both time efficiency and patient satisfaction in their clinical work while maintaining medical quality.

## Conclusion

The analysis revealed different behavioral strategies and communicative skills that contributed to time efficiency and patient satisfaction while maintaining medical quality. The three most important behaviors for time efficiency were GP continuity, ability to handle interruptions, and good administrative strategy. For medical quality, it was especially important that the GP explored the patient’s agenda, concerns, and symptoms, focused on performing one task at a time, and could acknowledge and learn from medical uncertainty. For patient satisfaction, GP continuity and relationship ranked most highly, supported by keeping the patient in focus and being able to switch between different communicative styles to connect with patients and fulfill their needs for care.

### Strengths and limitations of the study

The study was designed to avoid creating unnecessary stress for the GPs that could have affected their normal work patterns. The GPs received information before the project started that the goal was to describe good work strategies and that they had been chosen to participate because they were known to be experienced and well-functioning in their respective PHCs. Although the participants were selected strategically to focus on the research issue, we still collected enough data to explore general, time-efficient, and effective work methods. The codes and categories were repeatedly observed with all participants with some new information added until the data was saturated. Overall, the information gathered from the participants was sufficient to answer the study’s questions.

The feedback of randomly chosen patients (not all of whom answered the phone and participated) was fairly evenly divided between positive and negative, and all was based on logical reasons well correlated with the observations. Limitation of the study was the small number of participants, which may reduce the transferability of the study results as these participants may not represent all well-functioning and experienced GPs. The size of the data collected from patient interviews were considerably smaller compared to the empirical data obtained from the observations, thus skewing the weight of the study more towards the results of the observations rather than the patient interviews being more transferable. Despite this, the results regarding the behavioral strategies may be important for other medical colleagues who are looking for effective methods to improve their clinical patient work.

### Future directions

To ensure the transferability of these results, this study needs to be repeated in a larger population, with the observations video-recorded and more than one observer to co-evaluate all observations. The result from the study could then be used in a possible future discussion in PHCs with GPs to address different working environmental issues.

There was feedback from patients expecting different communication styles from the GPs, stressing the importance of remembering to ask for the patient’s expectations, which should be interesting to study in the future.

Other potential gains could come from expanding the method to study professionalism in other medical health care sectors where the described behavioral strategies and communicative skills may also have an important impact on improving goal-oriented conversations for better patient satisfaction, quality of care, and time efficiency.

## References

[1] Swartling P. Den svenska allmänmedicinens historia. [The history of Swedish general medicine]. Läkartidningen. 2006;103:1950–1953. Available at: https://lakartidningen.se/wp-content/uploads/OldWebArticlePdf/4/4318/LKT0624s1950_1953.pdf (accessed 6 September 2020). 16838581

[2] Socialstyrelsen. Primärvårdens uppdrag [The mission of primary care]. 2016. Available at: https://www.socialstyrelsen.se/globalassets/sharepoint-dokument/artikelkatalog/ovrigt/2016-3-2.pdf. (accessed 6 September 2020).

[3] Lindhagen K. Om allmänmedicin för allmänheten [About general medicine for the general public]. SFAM, 2014. Available at: http://sfam.se/foreningen/om-sfam/om-allmanmedicin-for-allmanheten/. (accessed 6 September 2020).

[4] Malterud K, Hunskår S. Svensk granskning och bearbetning: redaktionskommittén [swedish review and processing: editorial committee], Article 31263. In: Hunskår S, Hovelius B (eds) Allmänmedicin [general medicine]., 2nd ed. [in Swedish], Wallgren GA, Jones L (trans). Studentlitteratur AB; 2007. rev2015 p 29–37.

[5] Byrne PS, Long BEL. Doctors talking to Patients. A Study of the Verbal Behaviour of General Practitioners Consulting in their Surgeries. London: Her Majesty’s Stationery Office, 1976.

[6] Svenska Distriktsläkarföreningen. Allmänläkarens uppdrag – och andras… om uppdragsväxling, kompetens och kompetensutnyttjande i primärvården [The general practitioner’s assignment - and others’ … on assignment exchange, competence and utilization of competence in primary care]. Available at: http://distriktslakaren.se/sites/default/files/Allmänläkarens_uppdrag_och_andras_Task_shifting_2014.pdf. (accessed 6 September 2020.

[7] Aronsson G, Beijerot E, Härenstam A. Onödiga och oskäliga arbetsuppgifter bland läkare [unnecessary and unreasonable tasks among doctors. Läkartidningen. 2012;48:2216–2219. Available at: https://lakartidningen.se/klinik-och-vetenskap-1/2012/11/onodiga-och-oskaliga-arbetsuppgifter-bland-lakare/ (accessed 7 September 2020). 23330528

[8] Rivera-Rodriguez AJ, Karsh B-T. Interruptions and distractions in health care: review and reappraisal. Qual Saf Health Care. 2010;19(4):304–312. 20378621 10.1136/qshc.2009.033282 PMC3007093

[9] Wedin M. Kartläggning visar primärvårdens brist på specialister [Surveys show primary care’s lack of specialists]. Läkartidningen. 2013;110:538–542. Available at: https://lakartidningen.se/opinion/signerat/2013/03/kartlaggning-visar-primarvardens-brist-pa-specialister/ (accessed 7 September 2020).

[10] Pettersson S, Jaktlund Å. Läkarförbundets undersökning av primärvårdens läkarbemanning [The Swedish Medical Association’s survey of primary care’s medical staff]. Sveriges läkarförbund; 2013. Available at: http://distriktslakaren.se/sites/default/files/Prim%C3%A4rv%C3%A5rdens_l%C3%A4karbemanning_final.pdf. (accessed 7 September 2020).

[11] Myndigheten för vård- och omsorgsanalys. Allmän tillgång? Ett kunskapsunderlag för en stärkt försörjning av läkarkompetens i första linjens vård [Public access? A knowledge base for a strengthened supply of medical comopetence in first-line care]. Report 2018:5. Available at: (accessed 4 February 2021;https://www.vardanalys.se/rapporter/allman-tillgang/

[12] Sveriges Riksdag. Regeringens proposition 2008/09:74, Vårdval i primärvården [Choice of care in primary care. Proposition 2008/09:74]. Published December 2008. updated April 2015. Available at: https://www.regeringen.se/rattsliga-dokument/proposition/2008/12/prop.-20080974/. (accessed 4 February 2021).

[13] Meryn S. Improving doctor-patient communication. Not an option, but a necessity. BMJ. 1998;316(7149):1922–1930. 9641926 10.1136/bmj.316.7149.1922 PMC1113402

[14] Pinto RZ, Ferreira ML, Oliveira VC, et al. Patient-centred communication is associated with positive therapeutic alliance: a systematic review. J Physiother. 2012;58(2):77–87. 22613237 10.1016/S1836-9553(12)70087-5

[15] Gronseth IM, Malterud K, Nilsen S. Why do doctors in Norway choose general practice and remain there? A qualitative study about motivational experiences. Published online: 12 May 2020. 10.1080/02813432.2020.1753348 (accessed 2022-01-11) PMC8570750 32396781

[16] Houwen J, Lucassen P, Stappers H How to learn skilled communication in primary care MUS consultations: a focus group study. Published online: 11 Feb, et al. 2021. 10.1080/02813432.2021.1882088 (accessed 2022-01-11) PMC7971340 33569982

[17] Eide BT, Straand J, Braend AM. Pages 296-304. Published online: 27 May 2021. (accessed 2022-02-21)

[18] Larsen J-H, Risør O, Putnam S. PRACTICAL: a step-by-step model for conducting the consultation in general practice. Fam Pract. 1997;14(4):295–301. 9283850 10.1093/fampra/14.4.295

[19] Nystrup J, Larsen J-H, Risør O. Developing communication skills for the general practice consultation process. Sultan Qaboos Univ Med J. 2010;10(3):318–325. 21509251 PMC3074725

[20] Larsen JH, Neighbour R. Five cards: a simple guide to beginning the consultation. Br J Gen Pract. 2014;64(620):150–151. 24567647 10.3399/bjgp14X677662 PMC3933835

[21] MINT. Understanding motivational interviewing. Available at: https://motivationalinterviewing.org/understanding-motivational-interviewing. (accessed 7 September 2020.

[22] Landström B, Rudebeck C, Mattsson B. Working behavior of competent general practitioners: personal styles and deliberate strategies. Scand J Prim Health Care. 2006;24(2):122–128. 16690562 10.1080/02813430500508355

[23] Landström B, Mattsson B, Rudebeck C. Attributes of competence – on GPs’ work performance in daily practice. Scand J Public Health. 2009;37(6):598–603. 19451198 10.1177/1403494809105433

[24] Patterson F, Ferguson E, Lane P, et al. A competency model for general practice: implications for selection, training, and development. Br J Gen Pract. 2000;50(452):188–193. 10750226 PMC1313648

[25] Recapturing time: a practical approach to time management for physicians. Postgrad Med J. 2014;90:267–272. 24599633 10.1136/postgradmedj-2013-132012

[26] Graneheim U, Lundman B. Qualitative content analysis in nursing research: concepts, procedures and measures to achieve trustworthiness. Nurse Educ Today. 2004;24(2):105–112. 14769454 10.1016/j.nedt.2003.10.001

[27] Kelley J, Kraft-Todd G, Schapira L, et al. The influence of the patient-clinician relationship on healthcare outcomes: a systematic review and meta-analysis of randomized controlled trials. PLoS One. Published Online. 2014;9(4):e94207. 10.1371/journal.pone.0094207 24718585 PMC3981763

